# Topics of Public Interest

**Published:** 1904-12

**Authors:** 


### TOPICS OF PUBLIC INTEREST.

### Unification Failure as Others See It.

[*Detroit Medical Journal*, November, 1904.]

THE long looked and hoped for union of the regular profession of New York State has been postponed for a year at least, because of the objection of a few members of the New York State Medical Association to the legality of the proposed union. This quibble looks from this distance like hair-splitting and it is much to be regretted that some way was not found to silence the dissenters to secure unanimous consent for the adoption of the new code of rules, regardless of any technical unconstitutionality, as in point of fact was done originally by the A. M. A. at the Saint Paul meeting and by many state societies since.

### The Obstruction to Corporate Medical Reunion in New York.

[*Saint Louis Medical Review*, November 19, 1904.]

THE schism that has existed for so many years in the medical profession of the state of New York, and which, by an agreement between committees specially appointed for the purpose by the rival societies,—the ancient Society of the State of New York, and the more youthful and prosperous New York State Medical Association, the offspring of the American Medical Association,—seemed practically healed by granulation, has again broken down from undue tension at its weakest spot. The Onondaga County Medical Association objects to the healing process, and has taken advantage of a little inattention to "secundum artem" methods, to refuse to do its share of epidermal extension. So, for a while, at least, the sore must remain open. The *New York State Journal of Medicine,* the official organ of the association, appears to have thrown up the sponge grafts, and to consider further healing efforts useless, and appeals to all physicians in good standing to come into its fold, holding out to them the right hand of fellowship, and adds: "Let those who are jealous to preserve the more aged society belong to both. Preserve the other, if you will, by a meeting now and then,—though, for ourselves, we are not antiquarian (some of us)."

While all difficulties of principle in the way of members of the Medical Society of the State of New York becoming members also, if they wish, of the New York State Medical Association, have been removed by the action of the American Medical Association at Atlantic City, that would scarcely effect the issue desired, viz.: the corporate reunion of the two bodies. A little patience will probably convince the Onondaga County Association, even yet, that for twelve members to oppose themselves to the healing process extending inwards from all sides, is a little suggestive of "proud flesh," needing surgical treatment.

Meanwhile, the preservation and extension of the "antiquarian" and historical spirit is just what some others of us, in growing numbers, believe to need extended cultivation in the interests of medicine and the profession.

---

### The Japanese Medical Department.

[*The Medical Age*, November 10, 1904.]

IN THE gigantic struggle which is at present going on in the Far East nothing but praise is heard of the Japanese in their manner of conducting their army and medical department. Major L. L. Seaman, who has just returned from the seat of war, in an address delivered before the Association of Military Surgeons of the United States said: "The medical officer is omnipresent. You will find him in countless places where, in an American or British army, he has no place. He is as much at the front as in the rear. He is with the advanced scouts, with his microscope and chemicals, testing and labeling wells so the army to follow shall drink no contaminated water. When the scouts reach a town he immediately institutes a thorough examination of its sanitary conditions, and if contagion or infection is found he quarantines and places a guard around the dangerous district. Notices are posted so the approaching column is warned, and no soldiers are billeted where danger exists. Microscopic blood tests are made in all fever cases, and bacteriologic experts, fully equipped, form part of the staff of every divisional headquarters. The medical officer is also found in camp, lecturing the men on sanitation and the hundred and one details of personal hygiene,—how to cook, to eat, and when not to drink, to bathe, and even to the direction of the paring and cleansing of the fingernails to prevent danger from bacteria."

---

### The State Medical Library.

[*Albany Medical Annals*, November, 1904.]

BY invitation of the director the State Medical Library was placed at the service of the Medical Society of the County of Albany on the evening of October 11, and the semiannual meeting was held there. Mr. Melvil Dewey, the director, who intended to be present, was kept away by sickness, and in his absence, Mr. Dunkin Van Rensselaer Johnston welcomed the society, and in a brief address presented clearly the purposes, claims and difficulties of the library. It is the good fortune of the *Annals* to publish Mr. Johnston's remarks in this issue, and the attention of its readers is urgently directed to what he said. It will be seen that organisation has been effected and a large number of books secured, under the wretchedly inadequate appropriation of thirty-two hundred dollars a year. It may be readily understood why the government should provide for its legislators and other officials a perfect law library, but it is somewhat difficult to comprehend why the medical department should have been so neglected. Accessibility of the best and latest literature should be easy to all physicians, and Mr. Johnston's hints may well be carried home to every practitioner. A united effort should be made by individuals and societies to impress upon the legislature proper recognition of their needs in respect to this library. Its availability and the facility of securing the use of books and periodicals have been announced regularly in the *Annals,* in a department established for its use during the present year and edited by Miss Bunnell, the medical librarian. Mr. Dewey's ideas have also been announced in a contribution published in the *Annals* of March, 1904, and in a letter to Dr. Boyd, the president of the society, he writes: "I wish you would say, if there is opportunity, to your associates, how keenly I am interested in this medical library and how earnestly I desire to help its development." Dr. Andrew S. Draper, state commissioner of education, the executive of all the departments controlled by the board of regents, addressed the society informally, expressed his interest in the medical library, and announced his desire to establish a "library on Capitol Hill second to none in this country." It should be understood that this library is not local, but is for the use of the physicians of the state of New York. In their efforts to develop it, the physicians of Albany act for the general good, and they should be heartily and energetically supported. The selection of the advisory committee consisting of Drs. Albert Vander Veer, Samuel B. Ward, Henry Hun, George E. Gorham and Arthur W. Elting, carries the assurance of a wise and discriminating use of whatever moneys are appropriated by the state.

---

### The Medical Service of the Panama Canal Zone.

[*New York Medical Journal,* November 19, 1904.]

In its issue for November the *Journal of the Association of Military Surgeons of the United States* gives the following list of medical officers thus far assigned to duty in the zone, with their annual salaries: Colonel William C. Gorgas, of the army, chief sanitary officer, $7,500; Medical Director John W. Ross, of the navy, director of hospitals, $7,000; Major Louis A. La Garde, of the army, superintendent of the canal hospital, $6,000; Surgeon Henry R. Carter, of the Public Health and Marine Hospital Service, chief quarantine officer, $5,750; Surgeon L. W. Spratling, of the navy, in charge of sanitary work, $5,250; Dr. A. B. Herrick, pathologist and clinician, $4,000; Captain A. N. Stark, of the army, physician, $3,600; Surgeon H. A. Stansfield, of the Public Health and Marine Hospital Service, in charge of the laboratory in Panama, $3,000; Mr. J. A. Le Prince, sanitary officer, $3,000; Lieutenant Theodore C. Lyster, of the army, executive officer, $2,400; Dr. Lewis Balch, health officer, $3,000; Dr. E. S. Wheeler, Dr. W. F. Smith, Dr. T. B. Wingo, and Dr. D. Lacroisade, assistant physicians, $2,400 each; and Dr. Lloyd Nolan, assistant physician, $1,500. Dr. J. C. Perry, Dr. C. C. Pierce, and Dr. F. W. Ames, all officers of the government services, have been appointed members of the medical staff, but their salaries have not yet been determined. It will be seen that this list includes the names of a number of well known sanitarians, some of them men of great distinction. Unless the salaries mentioned are additional to their regular pay as members of the government medical services, we think they are for the most part inadequate, for much responsibility will go along with their duties, and even their great abilities will be taxed in the work that they will have to perform under novel circumstances.

In the same number of the *Journal,* Medical Director Ross gives an interesting account of the sanitary resources of the zone, present and prospective. There is the Ancon Hospital, at the Panama end of the zone, with a capacity of about 500 beds. Its buildings are to be repaired and modernised, and a training school for nurses is to be established in connection with the hospital. In this hospital will be stationed those of the Sisters of the Order of Saint Vincent de Paul previously connected with it, with the Strangers' Hospital (lately absorbed by the Ancon), and with the Colon Hospital as may elect to remain in the isthmian service under the new order of things. The Colon Hospital, formed by the consolidation of the French Canal Company's hospital and that of the Panama Railroad Company, has a present capacity of 100 beds, which will at once be enlarged 50 per cent. Along the line of the canal there will be a number of emergency hospitals and dispensaries. Two of these hospitals have already been started, one at Culebra and one at Gorgona. They are of the capacity of eight beds each. Two new dispensaries, under Dr. J. P. Bates and Dr. H. T. Summergill respectively, are to be established immediately. The municipal hospital of Panama, Santo Tomas, which is said to be defective in construction and heretofore wretchedly managed, is to be thoroughly put in order according to modern equipment methods. There is an ill conditioned leper colony on the outskirts of Panama. It is thought that within a short time a suitable and permanent institution for the care of lepers will be established. Heretofore the insane of Panama have been kept in the city prison, but lunatics are now cared for temporarily in buildings within the grounds of the Ancon Hospital, and preparations are in progress for the construction of a building large enough to accommodate the insane of both the zone and the Republic of Panama. There is a convalescent station, established by the French, on the salubrious island of Taboga, capable of accommodating about 150 patients. It is to be put into a suitable condition of repair without delay. It is easy to realise from these data that the work of our medical officers in the zone is to be of no trifling scope or extent. There will be ample opportunities for the display of their best efforts.

---

### New York State Hospitals for the Insane.

An abstract of the reports of the lunacy commission for the last three years has been made, showing the progress in the care of the insane. This commission is a constitutional body, consisting of one democrat, one republican and one independent, appointed by the governor and confirmed by the senate.

The chief objects accomplished by the commission in lunacy may be summarised as follows:

1. The reorganisation of the pathological institute.
2. Provision for the addition of 5,147 beds to the capacity of the present hospitals.
3. The segregation of tuberculous insane, at first in solariums and tents at the various hospitals, and later by the construction of three tuberculosis hospitals for 100 each.
4. The securing of an appropriation for the construction of ten isolation pavilions for infectious diseases.
5. The passage of a law providing for emergency commitment of serious cases of insanity.
6. Large additions to the means of treatment in asylums, in the way of surgical operating rooms, hydrotherapeutic apparatus and numerous electrical, medical and surgical appliances.
7. The introduction of a system of careful registration of each patient restrained or isolated, which has resulted in a large diminution in restraint of patients by mechanical means, and the like, and in the reduction of the number kept in solitary seclusion.

8. Throwing open the 14 state hospitals to 30 clinical assistants, who have the same opportunities for study, and for giving the same benefits to these institutions, as the similar arrangement for medical internes in our general hospitals.

9. A marked increase in the number of alien insane deported, and improving the facilities for discovering and deporting them.

10. The adoption of a more satisfactory dietary and larger ration than that allowed under the Atwater system.

11. The appointment of a medical inspector for the more continuous supervision of the 33 private asylums of the state in which about 1,000 patients are cared for.

12. Numerous systematic improvements demanded in such private retreats, as did not approach the standard of care set by a general letter sent out by the commission to the private asylums in January, 1902.

13. The development of a definite policy in the matter of provision for care of the insane by the state, which should be applicable not only now but for future years.

14. The appointment of boards of consulting specialists at a number of state hospitals, located sufficiently near to cities, resulting in great benefits to the institutions concerned.

15. The establishment of a summer colony on the lake shore for convalescent and curable patients, in connection with the Rochester State Hospital.

16. The establishment, now assured by the legislative authorities, of a colony for the insane in the Champlain region on the same principle as the Craig Colony for Epileptics, and of a psychopathic hospital in the city of New York.

A new pathological laboratory has been established in connection with the hospital on Ward's Island, where original work is going on in various departments of medical science, relating to psychiatry, and in addition to this, a majority of all the physicians of the fourteen state hospitals have been brought down to Ward's Island to receive instructions in modern science as relating to psychology, pathology and the treatment of insanity.

Particular insistence has been paid to the careful study of the clinical side of the patients admitted to the state hospitals. The histories of the patients now taken, not only present a clear picture of the mental condition of the patients, but record so completely every physical abnormality, that they are becoming a treasurehouse of important data for reference.

The practice of having regular staff meetings of the medical men once or twice weekly in each hospital, for the discussion of the conditions of the patients and for the presentation of new and interesting data in the domain of medicine, has been fostered. To this end, the commission has been liberal in the allowance of medical books and periodicals of all languages, in the purchase of instruments of precision, and in the establishment of well-equipped laboratories, surgical operating rooms, and departments of therapy and hydrotherapy.

The adoption of a definite policy for the further evolution of the state system of caring for the insane has been accomplished. The rapid growth of the state in population, and the constant influx of alien and nonresident insane, together with the normal accumulation of insane dependents, must inevitably lead to a greater demand for a period of years at least for increased accommodations for the insane. There are now about 25,000 insane in the state and 5,147 new beds have been added during the past two or three years.

The medical service has been improved by greater care in the selection of medical officers; and within a short time some new regulations will have been agreed upon with the civil service commission, which will enable the hospitals to secure a larger supply and better quality of medical assistants, and all of these men will have an opportunity to study in a special school of psychiatry connected with the pathological institute.

It is a pleasure to record as above the progress and development of the state's care of the insane during the past three years.

George B. Fowler, M.D.,
Consulting Physician to Bellevue and the French Hospital,
Former Commissioner of Health, New York City.

Edward D. Fisher, M.D.,
Professor of Nervous Diseases, Bellevue and
University Medical College.

Carlos MacDonald, M.D.,
Former President State Commission in Lunacy, Consulting Physician to the Manhattan State Hospital,
Professor of Mental Diseases in the University and
Bellevue Hospital Medical College.

---

### The Validity of an Agreement Not to Practise in a Given Locality.

[*Medicine*, November, 1904.]

A physician of Atlantic City (*Medical Record,* Aug. 20, 1904,) has filed a bill in chancery to show cause why another physician should not be enjoined from further practice of medicine in that city. The bill sets forth that the complainant has been practising medicine in Atlantic City for more than twenty-five years, and that during the last fifteen years he has required the services of an assistant. During these fifteen years he has had numerous assistants, and he states that it was his invariable custom to require them to sign an agreement by the terms of which they deprived themselves of the right to practise medicine in Atlantic City after severing their connection with him. In March, 1901, he employed an assistant, making the same agreement with him. This assistant is now practising independently in violation of the agreement. and an injunction is asked to enforce the terms of the contract.

The outcome of this suit is worth watching. If it should be established that such a contract is valid it would do much to place the relation of principal and assistant upon a secure foundation, to the obvious advantage of both. Many practitioners would be glad to have an assistant, but they do not relish the possibility of having him ingratiate himself among his patients and then settle down in the immediate neighborhood and begin active competition. Practically all of our recent graduates, especially those without hospital training, would be greatly benefited by serving for a number of years as assistant to an older practitioner. In that way they would gain experience and that practical insight into their duties which the medical school and hospital training can only in part afford. Legal security that would protect the practitioner against unfair competition later would beget confidence in this relation and lead to its far more frequent employment. An assistant would often enable a practitioner in the less busy season of the year to obtain a vacation, to attend medical societies, or follow postgraduate study.

While not attempting to pass on the merits of the particular case cited, many advantages would grow out of such contracts if they are held to be equitable.

---

### The Spleen Not a Physiologic Necessity.

As TO the very important part which many physiologists attach to the spleen, Dr. J. Henry Carstens, of Detroit, Mich., in speaking before the Mississippi Valley Medical Association recently said that he had extirpated the spleen in two men. In both cases there was great debility and emaciation, and the operation disclosed sarcoma and was followed by complete recovery. In one case the patient gained ninety-six pounds in weight in the fifteen months following his discharge from the hospital. This would indicate that the spleen is not such an absolute necessity after all. Dr. Carstens's experience coincides with that of several other surgeons.—*Southern Cal. Practitioner,* November, 1904.

